# Habituation of visual adaptation

**DOI:** 10.1038/srep19152

**Published:** 2016-01-07

**Authors:** Xue Dong, Yi Gao, Lili Lv, Min Bao

**Affiliations:** 1 Key Laboratory of Behavioral Science, Institute of Psychology, Chinese Academy of Sciences, Beijing 100101, P.R. China; 2 State Key Laboratory of Brain and Cognitive Sciences, Beijing 100101, China; 3 University of Chinese Academy of Sciences.

## Abstract

Our sensory system adjusts its function driven by both shorter-term (e.g. adaptation) and longer-term (e.g. learning) experiences. Most past adaptation literature focuses on short-term adaptation. Only recently researchers have begun to investigate how adaptation changes over a span of days. This question is important, since in real life many environmental changes stretch over multiple days or longer. However, the answer to the question remains largely unclear. Here we addressed this issue by tracking perceptual bias (also known as aftereffect) induced by motion or contrast adaptation across multiple daily adaptation sessions. Aftereffects were measured every day after adaptation, which corresponded to the degree of adaptation on each day. For passively viewed adapters, repeated adaptation attenuated aftereffects. Once adapters were presented with an attentional task, aftereffects could either reduce for easy tasks, or initially show an increase followed by a later decrease for demanding tasks. Quantitative analysis of the decay rates in contrast adaptation showed that repeated exposure of the adapter appeared to be equivalent to adaptation to a weaker stimulus. These results suggest that both attention and a non-attentional habituation-like mechanism jointly determine how adaptation develops across multiple daily sessions.

The visual system adjusts to changes in the environment^1^. For example, prolonged viewing of a high contrast stimulus reduces both perceptual and neural sensitivity to subsequent stimuli of similar pattern^2^,^3^. Such adaptation is ubiquitous in the nervous system, making it important to study.

Most past work on visual adaptation has adapted subjects for relatively short periods ranging from minutes to hours^4^,^5^,^6^,^7^,^8^. However, in real life many environmental changes are long lasting. An example occurred around 1999, at which time flat screen monitors gradually began to replace spherical CRT monitors. People starting to use computers before that time may still remember that a flat screen monitor appears concave when first used. Such a nonveridical perception fades slowly over time as people get more and more accustomed to a flat screen. This and other such cases have spurred research on longer-term adaptation. Although it has been reported that effects of adaptation get stronger and longer-lasting as the adaptation duration lengthens^9^,^10^, it remains largely unknown how adaptation changes across multiple days. That is, at issue is whether adaptation is affected by previous experience with the adapting stimulus in the case of adaptation over multiple daily sessions.

To our knowledge, only a few recent studies have begun to investigate this issue^11^,^12^,^13^,^14^. The answer to this question so far is mixed. Yehezkel and colleagues found that the adaptation effect to four hours of simulated monocular astigmatism increased over two training days^11^. By investigating adaptation to other altered visual reality, however, Haak *et al.*’s work suggested a more complex picture^14^. Their subjects viewed a world lacking vertical information for 4 days continuously. Effects of adaptation on apparent contrast during the first day increased as a function of exposure time, but then showed a drop in strength followed by a slower increase from day 2 through day 4. In spite of these generally common findings in prism and contrast adaptation, repeated motion adaptation has been found to have no effect on the duration of motion aftereffect^12^,^13^. Given the use of distinct adapting stimuli (natural vs. artificial) and visual features or properties (blur, contrast vs. motion), it is difficult to understand what causes these conflicting findings. Therefore, the present study systematically addresses this issue with deliberate controls on such confounding factors. Two sets of experiments separately tracked perceptual bias (aka. aftereffect) induced by motion or contrast adaptation across multiple daily adaptation sessions. Perceptual bias was measured every day after adaptation, which corresponded to the degree of adaptation on each day. In the present study, subjects were repeatedly adapted at the same locations for over several days. We term these locations the exposed locations, and other testing locations the unexposed control locations. Though some researchers use the word *perceptual learning* to describe or interpret their repeated adaptation studies^11^,^13^, it may cause confusion to readers who favor using the term to refer only to practice-based learning^15^. Therefore, the observed effects in the present study will be instead termed simply *learning*, a more general and conservative word. And for simplicity, we use the word *training* to describe our repeated adaptation paradigms in this study.

## Results

### Repeated adaptation to motion in a given direction

**Figure 1:**
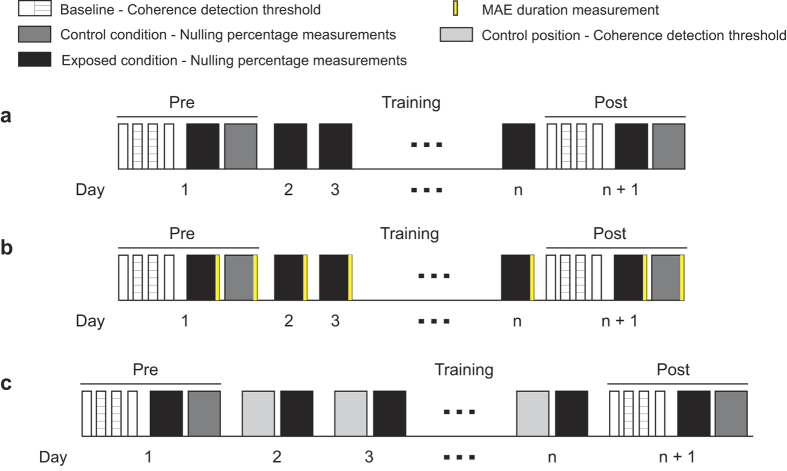
Flowcharts for the motion adaptation experiments . (**a**) The experimental protocol in Experiments 1.1 and 1.2. Four sessions were first completed to measure coherence detection thresholds at both the exposed (striped bars) and control locations (white bars) in the pre- and post-tests. The sequence was counter-balanced across the subjects. Then nulling percentages were measured under top-up adaptation. This was done by measuring the nulling percentages for adaptation to upward motion at one location and adaptation to downward motion at another location. During training, the nulling percentages were measured every day for adaption to motion in a fixed direction at a fixed location. The detailed procedure within each session was shown in Fig. 2a. The procedures for coherence detection measurements were similar except that the adapters were incoherently moving dots. (**b**) MAE duration was measured immediately after each block of top-up adaptation for 10 of the subjects in Experiment 1.1. (**c**) The experimental protocol in Experiment 1.3. On each training day, subjects completed both an adaptation session at the exposed location and an unadapted coherence detection session at the control location.

After viewing a moving stimulus for a period of time, either a stationary^5^,^16^,^17^,^18^ or a dynamic^19^,^20^,^21^,^22^ test pattern appears to move in the opposite direction to the original stimulus. This illusory motion is called the motion aftereffect (MAE). One way to estimate the strength of the MAE is to measure its duration^12^,^13^,^23^,^24^. However, this measurement is susceptible to such confounding factors as attention^25^,^26^,^27^. Moreover, the MAE measured with a static test pattern (i.e. static MAE) is considered to be mechanically different from that tested with dynamic stimuli^19^,^28^. For example, the former can be easily distinguished from real physical motion, but the latter cannot^19^. Therefore, although the previous literature fails to observe training-induced changes on the duration of static MAE, it remains likely that repeated adaptation still modifies the degree of motion adaptation in some aspects. Our first experiment estimated the strength of motion adaptation before and after 8–10 days of repeated adaptation to motion in a specified direction. The test stimulus was a random dot dynamic display. The method we adopted to measure this dynamic MAE was first introduced by Blake and Hiris (1993), where MAE can be perceptually nulled by a certain percentage of dots that consistently move in a direction opposite to that of the MAE^29^. For ease of more direct comparison with the previous literature, we also measured the duration of static MAE in 10 of the subjects. A flowchart for the experimental procedure is shown in Fig. 1.

### Repeated adaptation attenuated dynamic MAE (Experiment 1.1)

**Figure 2:**
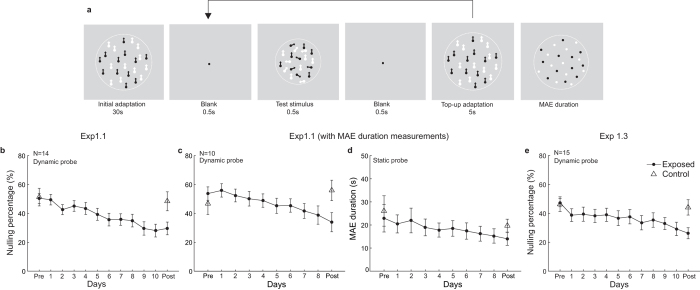
Procedure and training curves for Experiments 1.1 and 1.3. (**a**) Adaptation procedure. Subjects judged whether the test stimulus was moving upward or downward. Adapters were presented initially for 30 s with top-ups between each trial. The duration of each top-up was 5 s in Experiments 1.1 and 1.3, but randomized between 2 s and 8 s in Experiment 1.2.1. Separated by a 0.5 s blank interval (with a fixation point), test stimuli were presented for 0.5 s which consisted of noise dots whose individual directions were random and signal dots moving in the direction opposite to the motion aftereffect (MAE). Nulling percentages, i.e. the percentage of signal dots used to perceptually null the MAE, were measured with one-down-one-up staircases. (**b,c**) Training curves for two groups of subjects who were or were not measured for duration of MAE in Experiment 1.1, respectively. (**d**) The timecourse of MAE duration. (**e**) Training curve for the subjects who practiced the coherence detection task at the control location. ‘●’ represented the grand average nulling percentage for the exposed condition. ‘△’ represented the grand average nulling percentage for the control condition. Error bars in all the figures represented standard error of mean.

14 subjects were trained for 10 days to adapt to coherent dot motion in a top-up manner. Another 10 subjects were similarly trained for 8 days, but the duration of static MAE was measured after each training block. Specifically, each training block began with an initial 30-s adaptation. Afterwards, each test trial was preceded by a 5-s re-adaptation (See Fig. 2a). The adapting motion direction (upward or downward) and location (left or right visual field) were fixed for each subject but counter-balanced across the subjects. Test stimuli were random moving dots presented at the adapting location, with a certain proportion of dots moving in the adapting direction. Subjects were required to make a two-alternative-forced-choice (2AFC) response to judge the global motion direction of the test stimuli. Their responses were used to adjust the percentage of coherent dots with a one-down-one-up staircase. The nulling percentage (percentage of signal dots needed to perceptually null the MAE) was defined by the mean of the last 6 reversals of the staircase, which corresponded to the strength of the motion adaptation^29^. In addition to the measurements for the exposed condition, before and after training, nulling percentages were also measured at a contralateral control location for adaptation to direction that was the reverse of the one subjects were exposed to. For the 10 subjects measured for MAE duration, a test stimulus contained a single frame of static dots was displayed at the end of each training block, giving rise to the illusory perception of motion in the direction opposite to the adapting direction (i.e. static MAE). Subjects indicated when this MAE had ended by pressing a key. The mean duration of the aftereffect for each adapting direction was calculated across 5 trials per session.

Previous literature suggests that nulling method is not very effective at low dot densities (e.g. 39 dots/deg^2^), and that the nulling percentage measured at low dot densities may be close to the coherence detection threshold, because signal dots added in the test stimulus are not efficiently combined or paired with the noise dots^20^,^30^. Therefore, the present study adopted a relatively high dot density (238 dots/deg^2^, a bit lower than 311 dots/deg^2^ used in Castet *et al.*’s 2002 work).

Training (i.e. repeated adaptation) caused a robust decrease in nulling percentage (See Fig. 2b). A 2 (session: pre vs. post) × 2 (adapter: exposed vs. control) repeated measurements ANOVA on the nulling percentage (the first 14 subjects) revealed a significant main effect of session (*F*(1, 13) = 29.33, *p* < 0.001) and nonsignificant main effect of adapter (*F*(1, 13) = 0.83, *p* = 0.38). Importantly, the interaction between session and adapter was significant (*F*(1, 13) = 13.57, *p* = 0.003), suggesting that nulling percentage significantly reduced after training for the exposed condition (*t*(13) = 5.12, *p* < 0.001, paired t-test) but little for the control condition (*t*(13) = 1.54, *p* = 0.147, transfer ratio: 16%, see Fig. 2b). A linear trend analysis on the training curve of the nulling percentage also showed a reliable decreasing trend over the 10 days (*t*(13) = 4.88, *p* < 0.001). 7 of these subjects were able to participate in a follow-up test after 2-3 months. The training effects still remained robust (follow-up vs. pre: exposed condition, *t*(6) = 3.56, *p* = 0.012, control condition, *t*(6) = 1.78, *p* = 0.13), with only a small decay (proportion of retention, 71.08%; follow-up vs. post: exposed condition, *t*(6) = 2.36, *p* = 0.056, control condition, *t*(6) = 2.24, *p* = 0.067; See Supplementary Fig. S2).

Similar results were observed in the 10 subjects who were also measured for the duration of MAE. There was a significant decreasing trend for the nulling percentage over the 8 training days (*t*(9) = 4.16, *p* = 0.003, see Fig. 2c). The reduced nulling percentage was also found only for the exposed condition (*t*(9) = 3.89, *p* = 0.004) but not the control (*t*(9) = 1.36, *p* = 0.21). However, the duration of the static MAE did not significantly change over training (See Fig. 2d, linear trend analysis: *t*(9) = 1.68, *p* = 0.13; Post vs. Pre: exposed condition, *t*(9) = 1.76, *p* = 0.11, control condition, *t*(9) = 1.08, *p* = 0.31).

### Learning to reallocate attention in time (Experiment 1.2)

Experiment 1.1 revealed that repeated motion adaptation significantly reduced dynamic MAE (though not static MAE). One possible explanation of the results is that the degree of adaptation reduced over training. Alternatively, the results may reflect the changed deployment of attention in time^31^,^32^, since the same top-up paradigm was always used for training. Given that the stimulus timing was regular and that the task-irrelevant adapters were potentially distracting due to close temporal proximity with the test, a useful strategy would be to pay more attention to the test stimuli and less attention to the adapters.

### Randomizing the duration of the adapters (Experiment 1.2.1)

To test whether the reduced dynamic MAE over training resulted from learning to attentively suppress the adapters, 24 subjects were trained with a modified top-up paradigm. The duration of the top-up adapter in each trial was changed from 5 s to a random length between 2 and 8 s. This modulation should discourage the strategy of engaging temporal attention, because the onsets of the test stimuli became largely unanticipated.

**Figure 3:**
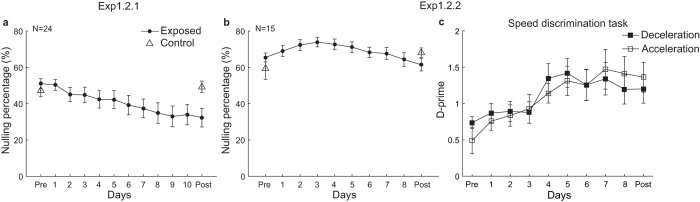
Training curves in Experiment 1.2. (**a**) The training curve in Experiment 1.2.1 (using random top-up duration). (**b**) The training curve in Experiment 1.2.2 where subjects performed a speed discrimination task on the adapter. (**c**) D-prime (Experiment 1.2.2) was calculated for the acceleration trials and deceleration trials, respectively.

Again, as shown in Fig. 3a, training with this modified top-up paradigm attenuated dynamic MAE (*t*(23) = 4.72, *p* < 0.001, linear trend analysis). The percentage of attenuation (37%) also approximated that (41%) in the original experiment (*t*(36) = 0.15, *p* = 0.87, two-sample t-test). The repeated measurements ANOVA showed resembling result pattern, with a significant session × adapter interaction (*F(*1, 23) = 15.78, *p* < 0.001) showing reliably reduced dynamic MAE only for the exposed condition (*t*(23) = 4.67, *p* < 0.001, paired t-test) but not for the control condition (*t*(23) = 0.82, *p* = 0.42).

These results speak against the attention hypothesis that subjects learn to selectively attend to the test intervals and suppress the adapters that are presented at a regular tempo. However, we cannot exclude a possibility that over training subjects still learn to ignore the adapters, and possibly wait for the adapter’s offset, whatever the top-up interval. Therefore, although the attention hypothesis is not advocated here, it is not definitely ruled out.

### Directing attention onto the adapters (Experiment 1.2.2)

To further examine the attention account, we trained 15 subjects to detect sudden acceleration or deceleration during each 6-s top-up adaptation. Other procedures and parameters resembled those in Experiment 1.1. Because subjects had to perform two demanding tasks together—a speed discrimination task during the top-up interval and a nulling task during the test interval—attention was believed to be focused on both the adapters and test stimuli. The goal of this experiment was to see how the dynamic MAE changed over training when the adapters were attended to. If attention is the unique account for the weakened aftereffects through repeated adaptation in Experiment 1.1, here directing attention onto the adapters should enhance the MAE^26^,^33^,^34^, or at least leave the MAE unchanged (when the effect of attentional enhancement is not strong enough).

Training significantly improved the speed discrimination performance for both acceleration and deceleration, as revealed by a linear trend analysis on the d-primes across sessions (acceleration: *t*(14) = 4.15, *p* < 0.001; deceleration: *t*(14) = 2.91, *p* < 0.05, see Fig. 3c). In direct comparison between the pre- and post-tests, the dynamic MAE was unchanged in either condition (exposed: *t*(14) = 1.33, *p* = 0.20; control: *t*(14) = 1.79, *p* = 0.096). However, an inspection of the training curve for the nulling percentage revealed more interesting information. There seemed to be an early increasing trend of the nulling percentage followed by a later decreasing trend. Piecewise linear regression indicated a break point of the training curve on the 3^rd^ training day (goodness of fit: 97.42%, highest among the candidate break points from the 2^nd^ through 7^th^ training day. Goodness of fit for all the candidate break points was 89.29%, 97.42%, 91.65, 80.89%, 67.66% and 58.34% respectively). As shown in Fig. 3b, there was a significant trend towards increased dynamic MAE through the pre-test day to the 3^rd^ training day (*t*(14) = 2.43, *p* = 0.029, linear trend analysis), hinting that attention might be a not-to-be-ignored factor contributing the changes in perceptual aftereffects in repeated adaptation. Remarkably, this increasing trend ended thereafter and showed a significant decreasing trend (*t*(14) = 3.43, *p* = 0.004).

The attention account could explain the increasing trend of the nulling percentage during the first few days, but cannot explain for this subsequent decline. This result suggests that attention may be not the unique mechanism mediating repeated motion adaptation. The accuracy for the speed discrimination task revealed that none of the difficulty levels resulted in performance hitting a ceiling. Except for the easiest acceleration condition, subjects’ accuracies remained below 80%, and even below 60% for the hardest acceleration condition. Thus there should be no reason for the subjects to attentively suppress the adapters over training. Whatever caused the later drop in the timecourse of the nulling percentage, it seems to be a mechanism distinct from attention.

Experiments 1.1 and 1.2 demonstrate that repeated adaptation to passively viewed random-dot motion attenuates dynamic MAE. When attention is directed to the random-dot adapter by a demanding task, the dynamic MAE shows a slightly increasing trend in the first few days followed by a gradual decrease over the remainder of training days. These results reveal a significant role played by attention, and also suggest a still-unexplained non-attentional mechanism that contributes to weaken the perceptual aftereffect. Previous studies on repeated motion adaptation fail to observe these training-induced changes on MAE. Since the adapter is passively viewed in one work^12^ and attended in the other^13^, the most likely reason for the divergence may not be attention itself but perhaps the way MAE was measured. Reporting the duration of MAE is essentially a Yes/No judgement on whether a stimulus is moving or not. This might be subject to bias and susceptible to attention. The nulling method we use requires a 2AFC report that may be less confounded by these factors. Another possibility is that Petrov *et al.*’s study measured the duration of MAE before and after 4 days of training where subjects only performed a direction discrimination task^13^, while our Experiment 1.2.2 trained the subjects for additional days. Thus Petrov and colleagues might not have the opportunity to detect any later changes.

### Concurrent training in the nulling task without adaptation at the control location (Experiment 1.3)

The general training effects we observed in the Experiments 1.1 and 1.2 resembled the effects observed in a set of repeated contrast adaptation experiments which will be introduced later. However, a dramatic difference was the amount of transfer. The training effects showed little transfer to the unexposed control condition in the motion adaptation experiments, but almost completely transferred in the contrast adaptation experiments (see Experiments 2.1 and 2.2). What caused this disparity?

We noticed one divergent procedural detail between these two sets of experiments. In the contrast experiments, training in the detection task may decrease baseline thresholds. This might produce unequal observed effects of adaptation between the exposed and unexposed locations. Therefore, in the Experiments 2.1 and 2.2, subjects were also asked to complete the same amount of detection trials at the unexposed control locations every day without adaptation. However, in the motion adaptation experiments, there was no training at the unexposed location. To test whether these different designs led to distinct transfer ratio, 15 subjects were trained as in our first motion adaptation experiment, but these subjects were also asked to do the nulling task *without* adaptation at the control location. As shown in Fig. 2e, the previously observed result pattern was replicated at the exposed location (*t*(14) = 4.78, *p* < 0.001), but still with little transfer (8.8%) of learning effects to the control location (*t*(14) = 0.42, *p* = 0.68). This suggests that concurrent training in an unadapted task at the control locations does not promote the transfer of learning effects from the exposed locations.

### Repeated adaptation to spatial contrast

**Figure 4:**
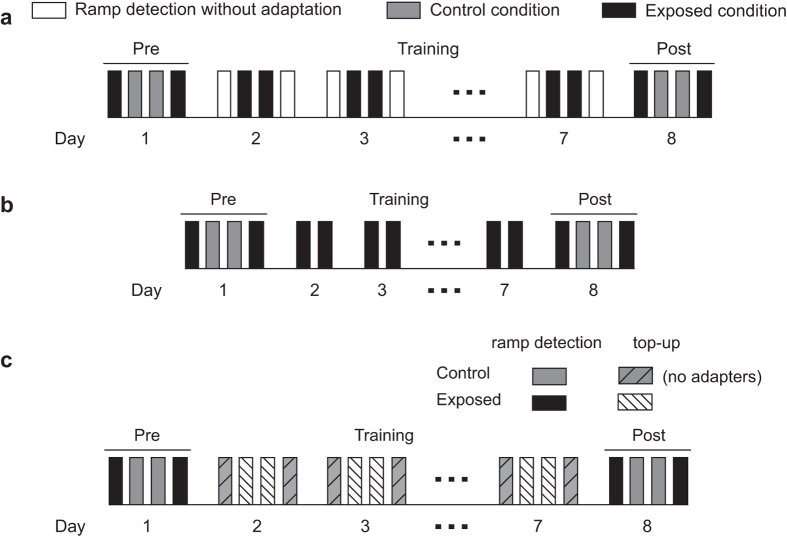
Flowcharts for the contrast experiments. (**a**) The experimental protocol in Experiments 2.1 and 2.2. Four sessions were completed in both the pre- and post-test day, with two in the exposed condition (black bars) and two in the control condition (gray bars). The session sequence was counter-balanced across the subjects. In the exposed condition, stimuli were presented at two diagonal positions (NW/SE or NE/SW) with one adapting orientation (vertical or horizontal). In the control condition, by contrast, stimuli were presented at the other two quadrants with the orthogonal orientation. The positions and orientation of the adapters remained the same within a session. During the 6 training days, subjects finished two adaptation sessions every day as in the pre- and post-tests but only for the exposed condition (black bars). Meanwhile, they completed another two unadapted sessions (white bars) where they were trained in ramp detection at the control positions with no adapters during the ‘adaptation’ phases. The detailed procedures for each session are depicted in Fig. 5a. (**b**) The experimental protocol in Experiment 2.3. The procedure was similar to Experiment 2.1, except that subjects were not trained in ramp detection at control locations during the training days. (**c**) The experimental protocol in Experiment 2.4. The pre- and post-tests were identical to the ramp detection paradigm in Experiment 2.2. However, a top-up paradigm was used for training. Subjects finished two adapted (at the exposed locations) and two unadapted (at the control locations) sessions every day. The detailed procedures for each session are depicted in Fig. 6a.

If attention and the elusive non-attentional mechanism jointly determine how effects of motion adaptation change over repeated adaptation, is this also true in repeated contrast adaptation ? Haak and colleagues (2014) found a general increase of contrast adaptation effect in subjects who lived for 4 days continuously in a world lacking vertical information^14^. Their “adapters” were not laboratory stimuli but the world around the subject that has been altered before being presented to the eyes as real-time videos. Undoubtedly, subjects always pay attention to the “adapters” when doing everyday activities in the environment of this altered reality. And thus attention may promote adaptation during the 4 days. To verify the role of attention and the non-attentional mechanism in repeated contrast adaptation using a method comparable to our motion adaptation experiments, we conducted another set of experiments adopting traditional psychophysical designs and laboratory stimuli. A secondary goal was to examine whether training affected the time course of aftereffect. With this aim we applied a modified method of limits we recently invented^35^, termed ‘ramp detection’, to measure the contrast aftereffect and track its decay. Specifically, the contrast of test gratings increased gradually from subthreshold to 50% within 3.5 s. Subjects were required to press a key as soon as they were just able to perceive the test gratings. The presentation of the test gratings was terminated once a key was pressed, or otherwise in 3.5 s. The stimulus contrast at the time of response was taken as a measure of detection threshold, corresponding to the contrast sensitivity at that time. A flowchart for the experimental procedure is shown in Fig. 4.

### Repeated adaptation reduced contrast aftereffect (Experiment 2.1)

**Figure 5:**
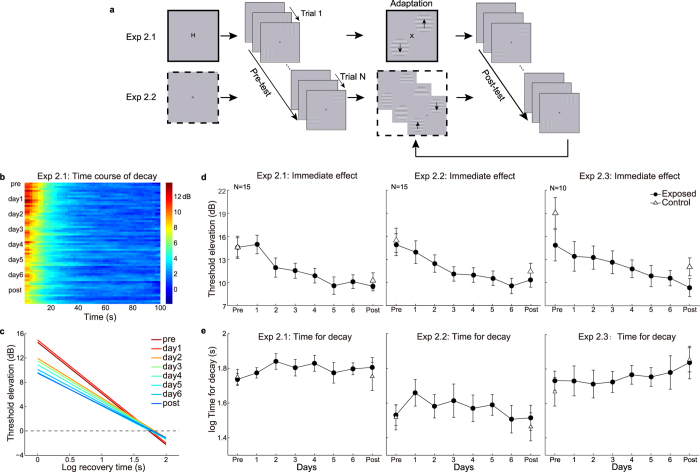
Procedures and results in Experiments 2.1, 2.2 and 2.3. (**a**) Two sessions were completed every day, each containing four adaptation-test cycles. Contrast thresholds were measured using a ramp detection method. The thresholds were tracked for 300 s before adaptation and after each 100-s adaptation phase. In each trial, subjects pressed a key when the test gratings were just visible. The contrast of the test gratings increased logarithmically at a constant rate. The solid and dash frames represent different attentional control during the adaptation phases for the two experiments—subjects performed a central RSVP letter task in Experiment 2.1, or monitored infrequent changes in spatial frequency of the adapters in Experiment 2.2. The arrows on the adapters represent that the adapters were drifting either towards or away from each other. Before the pre-adaptation tests, subjects also performed the RSVP task without adapters in Experiment 2.1, or just stared at the fixation point in Experiment 2.2. (**b**) Threshold elevation aftereffect (TEAE) in unit of dB was calculated by the formula: 
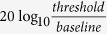
. Each row delineated the time course of TEAE for a single test phase. (**c**) Grand average fits of the decay function for TEAE in Experiment 2.1. The dash line represents the baseline estimated in each corresponding pre-adaptation test. (**d**) Training curves of the immediate TEAE. (**e**) The time for the adaptation effects to decay to the baseline on each training day.

Subjects performed a rapid serial visual presentation (RSVP) letter task for 100 s at the center of the screen while adapting to high contrast gratings drifted at two diagonal quadrants (See Fig. 5a). In the exposed condition, adapters were presented at two diagonal positions (NW/SE or NE/SW) with one adapting orientation (vertical or horizontal). While in the control condition, adapters were presented on the other two quadrants with the orthogonal orientation. The positions and the orientation of adapters remained the same within a testing session. This adaptation phase was followed by a 100 s testing phase, during which two test gratings were displayed in each trial at the adapting locations. The test gratings increased contrast at a constant rate from an initial invisible subthreshold level. Subjects were required to respond when the test gratings were just visible, which terminated the gratings’ presentation (and later presented the test gratings for the next trial). The stimulus contrast at the time of response was taken as a measure of detection threshold, corresponding to the contrast sensitivity at that time. Adaptation to high contrast led to elevated detection thresholds, a phenomenon often termed ‘threshold elevation aftereffect’ (TEAE). During the test phase, such contrast aftereffect gradually decayed towards the unadapted baseline level.

15 subjects were trained with this task for 6 days. To analyze the immediate TEAE, estimated at 1 s after the cease of adaptation phase, we performed a 2 (session: pre vs. post) × 2 (adapters: exposed vs. control) repeated measurements ANOVA. Only a significant main effect of session (*F*(1, 14) = 71.93, *p* < 0.001) was observed. Neither the main effect of adapters (*F*(1, 14) = 0.083, *p* = 0.777) nor the interaction (*F*(1, 14) = 0.005, *p* = 0.946) was significant. Paired t-test (pre- vs. post-test) indicated that the immediate TEAE significantly reduced for both the exposed (*t*(14) = 4.69, *p* < 0.001, see the left panel of Fig. 5d) and unexposed control adapters (*t*(14) = 6.19, *p* < 0.001). The transfer ratio was 102%. This suggested that training on contrast adaptation resulted in less degree of contrast adaptation, and this training effect could completely transfer to the unexposed condition. Interestingly, similar results were found in the slope of decay function (session: *F*(1, 14) = 52.71, *p* < 0.001, adapters: *F*(1, 14) = 0.29, *p* = 0.60, interaction: *F*(1, 14) = 0.30, *p* = 0.59). The slope of decay function became smaller after training (exposed adapter: *t*(14) = 5.33, *p* < 0.001, control adapter: *t*(14) = 4.96, *p* < 0.001, transfer ratio: 86%, see Supplementary Fig. S3a), while the estimated time for complete decay remained unaltered (exposed adapter: *t*(14) = 0.80, *p* = 0.43, unexposed adapter: *t*(14) = 0.03, *p* = 0.98, see the fitting lines for the exposed adapter on a log-log axis shown in Fig. 5c and the left panel of Fig. 5e). The linear trend analyses confirmed the training effects on the immediate TEAE (*t*(14) = 8.99, *p* < 0.001) and slope of decay (*t*(14) = 9.72, *p* < 0.001) progressively developed over training.

### Learning to reallocate spatial attention (Experiment 2.2)

Reminiscent of the concern of attention learning in our motion adaptation experiments is an argument that our observed reduction of TEAE may be a consequence of learning to ignore the task-irrelevant adapters rather than reduced degree of contrast adaptation. Therefore, another 15 subjects were trained with a modified paradigm where subjects were asked to monitor the occasional change of spatial frequency that occurred only at one of the two adapters. The attention learning account would expect spatial attention to improve over training at the exposed locations, which in turn might be expected to lead to enhanced contrast adaptation.

However, we observed result patterns very similar to the first contrast adaptation experiment (ANOVA for the immediate TEAE, session: *F*(1, 14) = 16.24, *p* = 0.001, adapters: *F*(1, 14) = 0.637, *p* = 0.44, interaction: *F*(1, 14) = 0.340, *p* = 0.57, see the middle panel in Fig. 5d; ANOVA for the slope of decay, session: *F*(1, 14) = 8.416, *p* = 0.012, adapters: *F*(1, 14) = 0. 612, *p* = 0.45, interaction: *F*(1, 14) = 0.543, *p* = 0.47.). Training still led to the reduction of the immediate TEAE and the slope of decay function in both the exposed (immediate TEAE: *t*(14) = 4.37, *p* < 0.001, slope of decay function: *t*(14) = 3.29, *p* = 0.005) and control conditions (immediate TEAE: *t*(14) = 2.55, *p* = 0.02, slope of decay function, *t*(14) = 2.07, *p* = 0.057), with a transfer ratio of 81% for the immediate TEAE and 78% for the slope of decay function. The estimated time for complete decay was also not affected by training (exposed adapter: *t*(14) = 1.48, *p* = 0.161, unexposed adapter: *t*(14) = 1.06, *p* = 0.31, see the middle panel in Fig. 5e).

Like Experiment 1.2.2, these results discredit the account that attention is the unique mechanism affecting repeated contrast adaptation. The training curve of contrast aftereffects may reflect the sum of signals of attention and a non-attentional mechanism. Over training the former tends to enhance adaptation, while the latter does the contrary. Whether the observed training curve is ascending or descending depends on which mechanism is stronger. When a more demanding task is used to direct attention to the adapter, the stronger training-induced enhancement can be produced by attention. As a result, training-induced attenuation due to the activity of the non-attentional mechanism may be counteracted or even overridden by attention. This supposition receives supports from the comparison between the performance of the attentional task in Experiment 1.2.2 and 2.2. As shown in Fig. 3c, training promoted the d-prime from about 0.74 to 1.20 for detecting deceleration and from 0.49 to 1.36 for detecting acceleration in Experiment 1.2.2, while in Experiment 2.2 the d-prime was increased from 2.80 to 4.36 (See Supplementary Fig. S4). Evidently, the attentional task in Experiment 2.2 is much easier than that in Experiment 1.2.2. This may explain why the perceptual aftereffect increased during the first few days in Experiment 1.2.2, however remained decreasing in Experiment 2.2. As to the later decline of the timecourse of aftereffect in Experiment 1.2.2, we speculate that after the first few days of training subjects became well-versed at the attentional task, and so the efficiency of attentional control might increase, leading to less attention signal involvement on subsequent days^36^,^37^. The signal of the non-attentional mechanism, therefore, turned to dominate the remainder of the timecourse.

### No training on contrast detection at the control locations (Experiment 2.3)

Following the logic similar to that in Experiment 1.3, we further examined whether training on contrast detection at the control locations caused the large transfer of learning effects. We replicated Experiment 2.1 in another 9 subjects who did not perform the baseline tests at the unexposed locations during the training. The results also resembled those in Experiment 2.1. As shown in the right panel of Fig. 5d, training significantly reduced the TEAE for the exposed condition (linear trend, *t*(8) = 4.21, *p* = 0.003; Immediate effect, Post vs. Pre: *t*(8) = 3.33, *p* = 0.010), and the effects also completely transferred to the control condition (Immediate effect, Post vs. Pre: *t*(8) = 2.75, *p* = 0.025). Training did not change the estimated time for complete decay in the exposed condition (*t*(8) = 1.77, *p* = 0.11, right panel in Fig. 5e). These results, together with the results of Experiment 1.3, exclude the explanation that training in an unadapted task at the control locations determines the transfer of learning effects from the exposed locations.

### Training of contrast adaptation in a top-up manner (Experiment 2.4)

**Figure 6:**
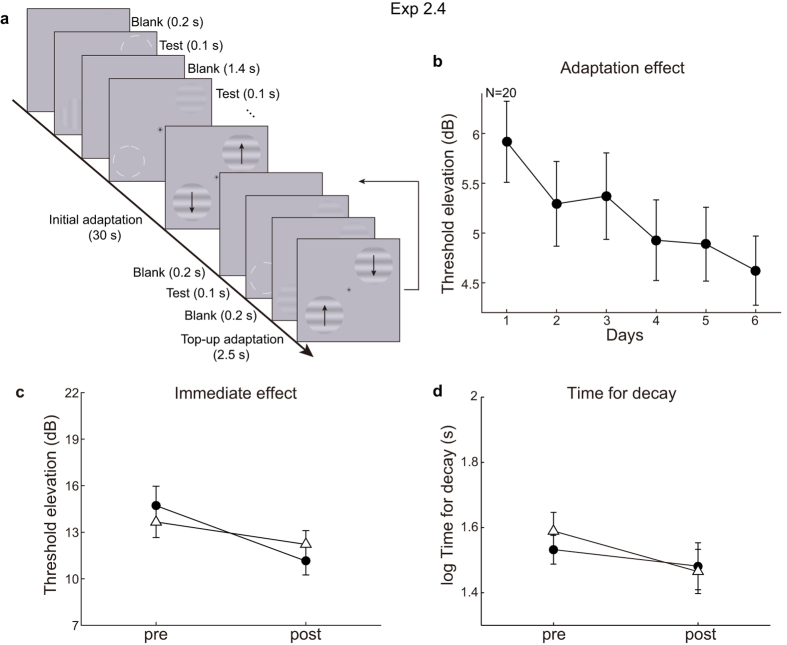
Training procedure and results in Experiment 2.4. (**a**) Training procedure in Experiment 2.4. Although subjects were pre- and post-tested with a ramp detection method, they were trained using a top-up paradigm as shown in this figure. Each training session started with a baseline measurement. Subjects were forced to indicate with a key press in which of the two locations a test grating appeared. The contrast of the test grating was modulated by QUEST in two interleaved staircases. Adapters were two drifting gratings that were initially presented for 30 s with 2.5-s top-ups between each trial. (**b**) Grand average training curve of the TEAE. 6 days of training significantly attenuated the adaptation effect. (**c**) The immediate TEAE measured in the pre- and post-tests revealed significant reduction after training for the exposed condition. However, the effects transferred only partially to the control condition. And this failed to reach statistical significance. (**d**) The time for decay measured in the pre- and post-tests.

Does the difference in transfer ratio reflect different traits between the two types of adaptation, or is it due to different training paradigms (top-up vs. continuous)? If the former is true, complete transfer should be replicated in repeated contrast adaptation with a top-up paradigm. We then ran an additional experiment on repeated contrast adaptation, but used a top-up training paradigm. 20 subjects were trained for 6 days on top-up contrast adaptation with testing trials using spatial 2AFC method (see Fig. 6a). For ease of comparison with the first two contrast adaptation experiments, we still used ramp detection for pre- and post-tests. For the exposed condition, training produced progressively decreased adaptation effect (linear trend analysis: *t*(19) = 4.49, *p* = 0.0002, Fig. 6b), and caused smaller immediate TEAE (*t*(19) = 3.13, *p* = 0.006, Fig. 6c) and slope of decay function (*t*(19) = 2.10, *p* = 0.0496) in the post-test than in the pre-test. The t-test revealed a significant difference of the time for decay in the control condition (*t*(19) = 2.22, *p* = 0.039) but not in exposed condition (*t*(19) = 1.02, *p* = 0.32, Fig. 6d). However, the transfer of learning effects to the control condition diminished (immediate TEAE: 42%, slope of decay function: 4.1%), and failed to reach statistical significance (immediate TEAE: *t*(19) = 1.55, *p* = 0.14, slope of decay function: *t*(19) = 0.08, *p* = 0.94). These results suggest that the complete transfer of training effects does not always occur in repeated contrast adaptation. Therefore, the amount of transfer cannot be simply ascribed to the type of adaptation (motion or contrast). Instead, it hints that for some reason using a top-up paradigm might correspond to less or no transfer of learning effects.

With three experiments (1.3, 2.3, and 2.4), what determines the amount of transfer in repeated adaptation remains an open question, although these results do serve to exclude two plausible explanations. Our observed training effects appear to be forms of learning (lasting more than 2-3 months), which at least contain an attention learning component and a non-attentional learning component. These effects certainly differ from typical perceptual learning or so-called practice-based learning^15^. However, we would like here to offer a conjecture based on the current opinions in perceptual learning, a more intensively studied field. Actually, specificity in perceptual learning is not ubiquitously observed^38^,^39^. A recent study suggests that training with difficult tasks promotes the specificity of perceptual learning^40^, supporting both the Reverse Hierarchy Theory^41^,^42^ and the Integrated Reweighting Theory^39^. If the same rules could apply for repeated adaptation, then task difficulty might be a factor contributing the distinct degree of transfer between our two sets of experiments. Based on the instruction, ramp detection threshold should be well above the chance level (50%) that the nulling method estimates. Therefore, ramp detection is believed to be an easier task as compared to nulling, this, in turn, likely generates more transfer in training. Since the performance in the Experiment 2.4 was set 82% correct by QUEST^43^ and a 2AFC method was used, the task here should be harder than ramp detection. And indeed, the training effects transferred less in the Experiment 2.4. However, since difficulty is being evaluated here across different tasks and types of adaptation, this explanation requires further investigation.

## Discussion

In 8 experiments, training in adaptation to motion or contrast was carried on for 6–10 consecutive days. We found that both types of adaptation effects tended to decrease over training. When attention was directed onto the adapter with a demanding task, this effect disappeared and might reappear later when the attentional task had been practiced for a few days. These results disclosed a novel form of learning under repeated adaptation that includes at least an attention learning component and a non-attentional mechanism.

Our results cannot be explained by a common learning effect on performing the perceptual bias task (e.g. nulling or detection) independent of adaptation. In the motion experiments, by definition coherence detection threshold has nothing to do with the nulling percentage. Thus training-induced changes on the latter are believed to be unrelated to the former. In the contrast experiments, training in the contrast detection task may improve contrast sensitivity to detect lower contrast gratings. If this improvement is more profound in the post-adaptation test, reduced TEAE would be observed. For example, subjects might be more tired in the post-adaptation test than in the pre-adaptation (i.e. baseline) test, thus showing poorer sensitivity following adaptation just because of fatigue. The TEAE could reflect both the adaptation and fatigue effects. It is not unlikely that the fatigue effects alleviate gradually across sessions, with the adaptation effects remaining unchanged. There could also be other factors instead of fatigue causing similar observations. However, such perceptual gain is not supposed to systematically change as a function of the time for decay. To put it in a mathematical way, on the log-log axis the learning of detection *per se* is expected to move the decay function parallel downward by a certain amount, and thus to cause shorter time required for the TEAE to reach the baseline as training proceeds. This is obviously at odds with our observations that the time for the TEAE to completely decay was largely unaffected by training (see Fig. 5c) while the immediate TEAE decreased across repeated daily sessions.

Nevertheless, this finding bears a striking resemblance to the manner in which adapting contrast modulates the time course of contrast adaptation. The literature reports indicate that the slope of decay functions increases as adapting contrast, but that the time required for thresholds to reach the baseline remains constant across different contrast levels^9^,^35^. It so seems that the effective strength of the adapter reduced after training, which in turn caused weaker aftereffects but left the time for decay unchanged. The reduction of adapter strength may result from habituation^44^. According to the Stimulus-Model Comparator theory of habituation^45^, with repeated experience of a stimulus, the nervous system creates a model of the expected stimulus. When the stimulus is presented again, it is compared with the stimulus model. Responding will be inhibited if the experienced stimulus matches the stimulus model. The stimulus model is usually not a very good representation of the presented stimulus initially, inhibition is thus weak at that time due to mismatching. However, with more repetitions, the stimulus model represents the stimulus more and more precisely, leading to gradually increased inhibition on the response. In the present study, repeated exposure to the same adapter over training may lead to such a stimulus model formed in the brain. As training proceeds, the stimulus model develops and as it develops, it exerts increasing descending inhibition to reduce the neural responses to the adapter. The outcome of exposure to the exposed adapter may thus be equivalent to the consequence of adaptation to a weaker stimulus. According to these clues, we think it very likely that habituation is the non-attentional mechanism causing reduced perceptual aftereffects over training.

Although we have reasoned in the Results section that our training effects should be a consequence of an interplay between attention and a non-attentional mechanism (probably habituation), Table 1 again shows why this is a more likely picture. Suppose attention is the only underlying mechanism. It should suppress the passively viewed adapters, and enhance the attended adapters. Results from Experiment 1.1 and 2.1 agree with the first prediction. However, the second prediction is not supported by the results of either Experiment 2.2 (where the attended adapters produced reduced aftereffects over training) or Experiment 1.2.2 (where the attended adapters produced increased aftereffects only during the first few days, but the aftereffects gradually decreased thereafter). If habituation alone determines the training effects, it cannot be explained why there was a significant trend towards increasing aftereffects during the first few days in Experiment 1.2.2. But if both mechanisms jointly work, with the sum of their signals determining the trend of training effects, all of our results can be explained. Over training attention may enhance adaptation, while habituation would reduce adaptation. Experiment 2.2 adopts a relatively easy attentional task. Therefore, the training effect of attention fails to override the effect of habituation. Experiment 1.2.2 uses a more difficult task, allowing the emergence of an early increasing trend of aftereffects. As the subjects became well-versed at the attentional task, the effect of attention might reduce. Thus the effect of habituation gains the dominance over the effect of attention. This may result in the decreasing trend in the later stage of training. It should be noted that the habituation account builds only upon the results of decay rates in the contrast adaptation experiments. Future work will give a more definite answer whether habituation also plays a role in repeated motion adaptation. In addition, neuroimaging techniques may also provide more direct measurements in response to this question.

Our work helps shed light on the mixed findings in the literature on how effects of adaptation change across repeated sessions^11^,^12^,^13^,^14^. Previous motion adaptation studies estimate the degree of adaptation based exclusively on the duration of MAE, which may be susceptible to attention and bias. Our method using nulling percentage is bias-free and benefits from greater testing powers (e.g. each session in Experiment 1.1 included 10 staircases, with each staircase consisting of about 30 trials). Indeed, we replicated the null results on MAE duration while concurrently measuring nulling percentages in the same experiment. Our results in Experiments 1.2.2 and 2.2 disclose, for the first time, the joint roles of attention and a habituation-like mechanism in mediating repeated adaptation. Previous observations of the increased effects of adaptation to altered visual reality^11^,^14^ might reflect the strong contribution of attention when people adapt to an environment that they live in.

In summary, our study explored how long-term experience modulated short-term experience-dependent plasticity. We found—for the first time, to our knowledge—that training could weaken the degree of both motion and contrast adaptation. This effect, however, may be compromised to some extent by directing attention to the adapting stimuli. Moreover, the way in which training altered the decay function of contrast aftereffects suggests that the apparent strength of adapters became weaker through training. This potentially relates such ‘adaptation learning’ to the biological phenomenon of habituation.

## Methods

All subjects gave informed consent prior to participation. Experimental procedures in the present study were approved by the Institutional Review Board of the Institute of Psychology, Chinese Academy of Sciences, and the work was carried out in accordance with the Code of Ethics of the World Medical Association.

### Part I: Motion adaptation experiments

#### Subjects

All subjects had corrected-to-normal vision. A total of 78 subjects (ages from 18 to 32) participated in the motion adaptation experiments, with 24 subjects (14 males) in Experiment 1.1; 39 subjects (19 males) in Experiment 1.2; and 15 subjects (7 males) in Experiment 1.3. All were naïve to the purpose of the study, except that the author Y.G. was a subject in Experiment 1.1.

#### Apparatus

Stimuli were presented on a gamma-calibrated Sony CRT monitor (1600 × 1200 pixels resolution at the refresh rate of 85 Hz), and programmed in MATLAB and Psychtoolbox. The mean luminance of the CRT monitor was 41.7 cd/m^2^. Subjects viewed the display at a distance of 1 m in a dark room. A chin-rest was used to minimize head motion.

#### Stimuli

All stimuli were presented on a mid-gray background, with a central fixation point (0.23°). Both the adapter (3000 dots, 238 dots/deg^2^, dot size: 0.056°) and test stimulus (1688 dots, same dot density and size) were displayed within an imaginary circular window (4° in diameter for the adapter and 3° for the test), centered 6° to the left or right of the fixation point. Half the dots were black, and the remainders were white. This was determined at random. In a motion sequence of the adapters, dots were initially randomly positioned in the circular window and then moved upward or downward coherently at a constant speed of 5 deg/s (if not elsewhere defined). Once the dots fell outside, they were wrapped to the opposite side of the window. Test stimulus consisted of signal dots which moved either upward or downward, and of noise dots whose individual directions were random (see Procedures for more details).

### Procedures

#### Experiment 1.1

##### Coherence detection threshold measurements

As reported in the literature^20^, nulling percentage is likely to be affected by coherence detection thresholds. Therefore, before and after training, we measured the unadapted coherence detection thresholds for upward and downward motion at the exposed and unexposed locations. This measurement was carried out prior to the adaptation sessions. Also, to ensure that subjects were familiar with the task at hand, they were permitted sufficient practice at the coherence detection task (4.14 ± 1.03 and 4.07 ± 1.14 sessions in the left and right visual field, respectively) before the formal experiments.

To control potential factors irrelevant to motion adaptation (e.g. stimulus timing and contrast adaptation), we introduced adapters consisting of incoherent moving dots when measuring the unadapted coherence detection thresholds. Each session began with 1 min of preparation during which only a central fixation point was presented on the mid-gray background. Afterwards, the subjects pressed a key to start the test. The incoherent motion adapters were presented initially for 30 s with 5 s re-adaptation (i.e. top-up) between each trial. In each trial, the top-up was followed by a 0.5 s blank interval. Then a test stimulus containing a certain percentage of signal dots was presented for 0.5 s. Subjects pressed the arrow key to indicate the perceived global moving direction of the test stimulus. The next trial came up 0.5 s after the cessation of the test stimulus.

The direction and percentage of signal dots of the probe was determined by two interleaved 2-down-1-up staircases in each session. These converged toward a probability of 71% to perceive a global motion in the upward and downward direction, respectively. In total, 43 levels of coherence percentage were predetermined for later use in the staircase. These increased logarithmically from 0.47% to 84.77%. Each staircase began with a coherence percentage of 46.03%. The step size for the staircase was initially 3 levels, and it decreased to 1 level after 4 reversals. The staircase would stop when it reached 8 reversals, or when 150 trials had been completed for a given session, whichever came first. The threshold of each staircase was calculated as the average of the last 6 reversals. Each location was measured for two sessions. The coherence detection threshold in a session was defined as half the difference between both the upward and downward thresholds. The mean threshold across the two sessions was taken as the starting level of coherence percentage in the nulling percentage measurements.

##### Nulling percentage measurements

Each testing session contained 5 blocks, with two staircases in each block. A 1 min rest was allowed between the blocks. The stimulus presentation resembled those for the coherence detection task except that the adapters were replaced with coherently moving dots (see Fig. 2a). The coherence percentages of probes were controlled by two interleaved 1-down-1-up staircases to perceptually null the MAE. For instance, if the adapting direction was upward, an upward response would decrease the percentage of signal dots, while a downward response would increase it. The initial percentage of signal dots was determined by the coherence threshold measured in baseline sessions, which remained the same for all the testing and training sessions. The step size was initially 16%. It decreased to 8% after the first reversal, and then to 4% after the second reversal. A block would end when 34 trials had been completed in each staircase. The mean of the last 6 reversals of each staircase was taken as the nulling percentage. Thus 10 nulling percentage data were acquired for averaging in each testing session. Each subject’s average nulling percentage for the exposed and control locations was then normalized by dividing by the mean nulling percentages for both conditions before training.

##### MAE duration measurements

Immediately after each block, a static display of dots was presented to measure the duration of MAE. Subjects indicated with a key press when the MAE had ended. In total there were 5 measurements in each session.

Training with the nulling percentage task spanned 10 consecutive days (8 days for the 10 subjects who were also measured for MAE durations). Each subject was assigned a fixed adapting direction (upward or downward) at a fixed adapting location (left or right visual field) for training. The assignment of the exposed adapting direction and location was random and counter-balanced across these subjects. Nulling percentages for adaptation to motion in the opposite direction at the contralateral location were also measured before and after training. This served as the unexposed control. During the training, subjects completed one session per day for the exposed condition only. However, they completed an exposed session and a control session in both the pre- and post-tests. The sequence of testing sessions was identical for each subject, but counter-balanced across subjects. Subjects were required to take a break for at least 45 min between successive sessions.

#### Experiment 1.2.1

Procedures in Experiment 1.2.1 were identical to Experiment 1.1 except that the duration of top-ups became unpredictable. This duration was randomly selected for each trial from 9 levels between 2 and 8 s in step size of 0.75 s. Also subjects practiced the coherence detection task sufficiently (5.17 ± 1.52 and 5.33 ± 1.40 sessions in the left and right visual field, respectively) before the formal experiments.

#### Experiment 1.2.2

Each adaptation session was cut down to 4 blocks since the duration of top-up adaptation was lengthened to 6 s. There was either a 0.5-s acceleration or deceleration during each top-up adaptation (for one subject, the acceleration was 20%, 30% or 40%, deceleration was 15%, 23% or 31% ; for the other fourteen subjects, the acceleration was 15%, 25% or 35%, deceleration was 10%, 18% or 26%). The onset of speed change occurred at a random timepoint between 1 s to 3.5 s after the onset of the adapter. Subjects were forced to indicate whether there was an acceleration or deceleration during each top-up adaptation period. In addition, they also needed to perform the nulling percentage task during the test interval.

D-prime was calculated for detecting acceleration and deceleration, respectively, with the error rate of acceleration (deceleration) serving as the false alarm rate of deceleration (acceleration). Linear trend analysis was used to test if the detection performance improved over training. Other analysis about nulling percentage resembled that in Experiment 1.1.

#### Experiment 1.3

The only procedural difference between Experiment 1.3 and Experiment 1.1 was that subjects were also trained to do the nulling task without adaptation at the control location.

### Part II: Contrast adaptation experiments

#### Subjects

All subjects had corrected-to-normal vision. In total, 59 subjects (ages from 19 to 26) participated in the contrast adaptation experiments, with 15 subjects (6 males) in Experiment 2.1, 15 subjects (8 males) in Experiment 2.2, 9 subjects (3 males) in Experiment 2.3, and 20 subjects (9 males) in Experiment 2.4. All were naïve to the purpose of the study, except that the author X.D. was a subject of Experiment 2.2.

#### Apparatus

Stimuli were presented on a gamma-corrected monitor (1024 × 768 @ 85 Hz). The display was driven by a Bits# 14-bit video card (Cambridge Research Systems) and calibrated with a Photo Research PR-655 spectrophotometer. To calibrate the display, we measured the luminance gamma curves and inverted them with a look-up table. All stimuli were viewed from a distance of 48 cm in a dark room, and a chin-rest was used to minimize head motion. The monitor mean luminance was 44.49 cd/m^2^.

#### Stimuli

All stimuli were sinusoidal gratings whose edges were smoothed with a Gaussian envelope. The spatial frequency of gratings was 1 cpd. Stimuli were presented in two diagonal quadrants (NW/SE or NE/SW), centered 5° away from the central fixation, and were always presented on a mid-gray background. Adapters were horizontal or vertical gratings of 29 dB (28.18%, calculated by 
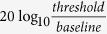
), which subtended 5° and drifted at a speed of 5 Hz (to avoid the formation of afterimages). The drifting direction was always orthogonal to the grating orientation, and the two gratings always drifted towards or away from each other. This may minimize eye movements. To avoid motion aftereffect, drifting direction changed to its opposite after every 1 to 5 s with the same total drifting duration for both directions. For the aim of Experiment 2.2, the spatial frequency of one of the two adapting gratings briefly (0.2 s) changed to 0.67 cpd at randomly selected moments.

Test gratings were either vertical or horizontal, subtending 3°. Contrast detection thresholds were measured using a ramp detection method^35^. The contrast of test gratings increased gradually from 0.1% to 50% within 3.5 s (15.42 dB/s). Subjects were required to press a key as soon as they were just able to perceive the test gratings. The presentation of the test gratings would be terminated once a key was pressed, or otherwise in 3.5 s. The stimulus contrast at the time of response was taken as a measure of detection threshold, corresponding to the contrast sensitivity at that time.

A central rapid serial visual presentation (RSVP) task was used to help maintain fixation during adaptation. A series of random capital letters were presented on the center of the screen (0.6°, each for 153ms). Subjects were required to detect the infrequent occurrences of letter ‘X’. While during the ramp detection, only a circle fixation (0.5°) was presented.

### Experiment 2.1

#### Procedure

Each session started with 100 s RSVP task followed by baseline measurements and four adaptation-test cycles. Contrast thresholds were tracked for 300 s before adaptation and after each 100-s adaptation phase using a ramp detection method. The last test phase was prolonged to 300 s in case the adaptation effect could not fully recover, though our previous work^35^ indicated that effects of adaptation for 100 s could completely decay within 100 s. In the adaptation phase, subjects performed the RSVP task while exposed to two drifting adapters. A brief tone was used to inform the subject of the upcoming ramp detection task before the end of adaptation.

Subjects finished the experiment in 8 successive days, with the first and the last day being devoted to the pre- and post-tests. Four sessions were completed each day in the pre- and post-test, two in the exposed condition and two in the control condition. The session sequence was counter-balanced. In the exposed condition, stimuli were presented at two diagonal positions (NW/SE or NE/SW) with one adapting orientation (vertical or horizontal). In the control condition, by contrast, stimuli were presented on the other two quadrants with the orthogonal orientation. The positions and the orientation of adapters remained the same within a testing session. During the 6 training days, subjects finished two adaptation sessions every day as in the pre- and post-tests but only in the exposed condition. Subjects also finished two unadapted sessions, in which they were also trained in ramp detection. This was done at the control positions with no adapters during the ‘adaptation’ phases. To reduce the residual aftereffects from previous sessions, subjects had to rest in a normal environment for an hour between two consecutive adaptation sessions.

Before the formal experiment, subjects finished on average 13 practice sessions (1.52 ± 0.52 h) consisting of 100 s RSVP task followed by 300 s of ramp detection, until they were familiar with the tasks and formed stable detection criterions. Subject’s detection criterion was considered stable as long as the standard deviation of thresholds measured in three successive practice sessions was less than 0.7% (See Supplementary Fig. S1a).

#### Analysis

Thresholds measured in the 4 test phases from a session were pooled together to create a single vector of time and threshold pairs. The thresholds measured before adaptation and in the last 200 s of the 4^th^ cycle were averaged to estimate a baseline threshold. Threshold elevation aftereffect (TEAE) in unit of dB was then calculated by the formula: 
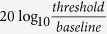
. The time course of decay was achieved by fitting the log time for decay and TEAE with a power function^9^,^35^. We first divided the test phases into time bins of constant widths on the log scale. This width was determined by the mean response intervals within the first 10 s after adaptation. The values within each time bin were then averaged. Before fitting on the pooled data across sessions, we needed to remove data points that already reached the baseline. Therefore, the binned time series in each session were nearest-neighbor interpolated, and then divided into several temporal groups from the end to the beginning of the time series. In most cases, each temporal group contained 3 data points. Data points from the same group of the same testing condition were compared to baseline with t-tests, allowing the data points that reached baseline to be excluded for fitting. The values at 1 s on the fitted regression lines were regarded as the immediate TEAE and the slope reflected the rate of decay. All the fitting results were normalized by dividing by the average immediate TEAE in the pre-test across the exposed and control conditions, which were then entered into the subsequent two-way repeated measurements ANOVAs, paired t-tests and linear trend analysis.

The performance of RSVP task was estimated with d-prime. Each target letter was first assigned to a 1.5 s time interval that started from the onset of the target. The rest time of the task period was also divided into 1.5-s intervals (An interval shorter than 0.75 s would be merged to the adjacent interval). Subjects’ responses were then categorized based on reaction times to calculate the d-primes. Responses within 1.5 s after the target onset were counted as hits, with those after 1.5 s as false alarms.

### Experiment 2.2

#### Procedure

The test and training procedures were similar to those in Experiment 2.1 except that each session started with viewing the mean field for 60 s, followed by a baseline measurement with ramp detection. Then the 4 adaptation-test cycles began. During the adaptation phases, subjects needed to monitor the infrequent changes in spatial frequency of the adapters. In the unadapted training sessions at the control positions, however, there were no adapters.

Similar to Experiment 2.1, before the formal experiment, subjects practiced the 300-s ramp detection task until they were familiar with the task and formed stable detection criterion (1.15 ± 0.26 h for naïve subjects, see Supplementary in Fig. S1b).

Analyses were the same as in Experiment 2.1.

### Experiment 2.3

The procedure was similar to that in Experiment 2.1 except that the ramp detection task was not practiced at the control locations during the training.

### Experiment 2.4

#### Procedure

The pre- and post-tests were identical to the ramp detection paradigm in Experiment 2.2. Top-up paradigm was used for training. TEAE was measured with a spatial 2-alternative-forced-choice (2AFC) method for top-up adaptation before and after training. Each session started with a baseline measurement. A trial began with a blank interval of 0.2 s, followed by a test grating appeared at one of the two diagonal locations for 0.1 s. Subjects were forced to indicate with a key press within 1.2 s in which of the two locations the test grating appeared. Adaptation began immediately after the baseline measurement. Two drifting gratings were presented simultaneously as in Experiments 2.1 and 2.2. They were initially presented for 30 s with 2.5-s top-ups between each trial. The contrast of test gratings was modulated by QUEST in two interleaved staircases (82% correct rate) for vertical and horizontal orientation, respectively. Each staircase contained 50 trials. Subjects finished two sessions for both the trained and control condition in each testing day. During training, subjects needed to finish two adaptation sessions in the exposed condition and two unadapted sessions at the control positions.

### Analysis

Analysis of ramp detection task was similar to Experiment 2.1. Data from four QUEST staircases in a training session were pooled together. A Weibull function was fitted to the data to estimate the contrast thresholds before and after adaptation.

## Additional Information

**How to cite this article**: Dong, X. *et al.* Habituation of visual adaptation. *Sci. Rep.* **6**, 19152; doi: 10.1038/srep19152 (2016).

## Supplementary Material

Supplementary Information

## Figures and Tables

**Table 1:** Predictions of three different accounts.

Which plays a dominant role in repeated adaptation? Attention or habituation?	Adapter is passively viewed		Adapter is attended with a demanding task	
	Prediction	Data	Prediction	Data
Only attention	AE reduces	Yes	AE increases	No
Only habituation	AE reduces	Yes	AE reduces	No
Both attention and habituation	AE reduces	Yes	All is possible	Yes

Note: AE represents perceptual aftereffects.
