# Supplementary material for: Habituation of visual adaptation
Source: Sci Rep. 2016 Jan 7;6:19152. doi: 10.1038/srep19152 (PMC4703959; doi:10.1038/srep19152)
Supplement: Supplementary Information [file srep19152-s1.pdf]

Supplementary Information

Habituation of visual adaptation

Xue Dong, Yi Gao, Lili Lv, Min Bao

Supplementary results of the baseline thresholds of contrast adaptation experiments

Experiment 2.1

As indicated by linear trend analysis, subjects' contrast sensitivity did not vary significantly during training (exposed condition: $t(14) = 0.33$, $p = 0.75$, control condition: $t(14) = 0.28$, $p = 0.78$, see the lower panel in Figure S3a).

Experiment 2.2

In both the exposed and control conditions, baseline thresholds decreased gradually over training (exposed condition: $t(14) = 3.93$, $p = 0.002$, control condition: $t(14) = 2.87$, $p = 0.01$, see the lower panel in Figure S3b). This suggested that subjects' contrast sensitivity improved over training. In Experiment 2.2, attention was always focused on the gratings. However, training on the central RSVP task in Experiment 2.1 might lead to suppression on the gratings, thus counteracted any improvement of neural gain in the periphery. Consistent with this hypothesis, the improved contrast sensitivity was only observed in Experiment 2.2. This result also agreed with the Attentional Weighting theory in perceptual learning[1].

Experiment 2.3

Linear trend analysis failed to disclose any change of contrast sensitivity during training in the exposed condition (See the lower panel in Figure S3c, $t(8) = 1.28$, $p = 0.24$). Baseline thresholds in the control condition were also unchanged ($t(8) = 0.40$, $p = 0.70$).

Experiment 2.4

The baseline thresholds in both the exposed and control conditions were not affected by training (See the lower panel in Figure S3d, exposed condition: $t(19) = 0.42$, $p = 0.68$, control condition: $t(19) = 1.27$, $p = 0.22$).

Supplemental Figures

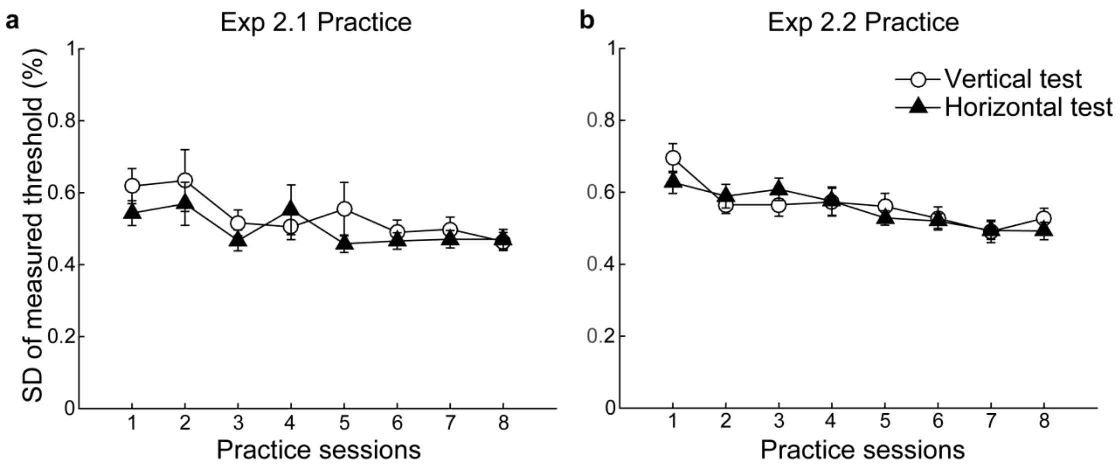

Figure S1. The performance in the practice sessions of Experiments 2.1 and 2.2. (a),(b) The standard deviation (SD) of the last eight practice sessions in Experiment 2.1 and 2.2. '○' denoted the SD for the vertical test, while '▲' for the horizontal test.

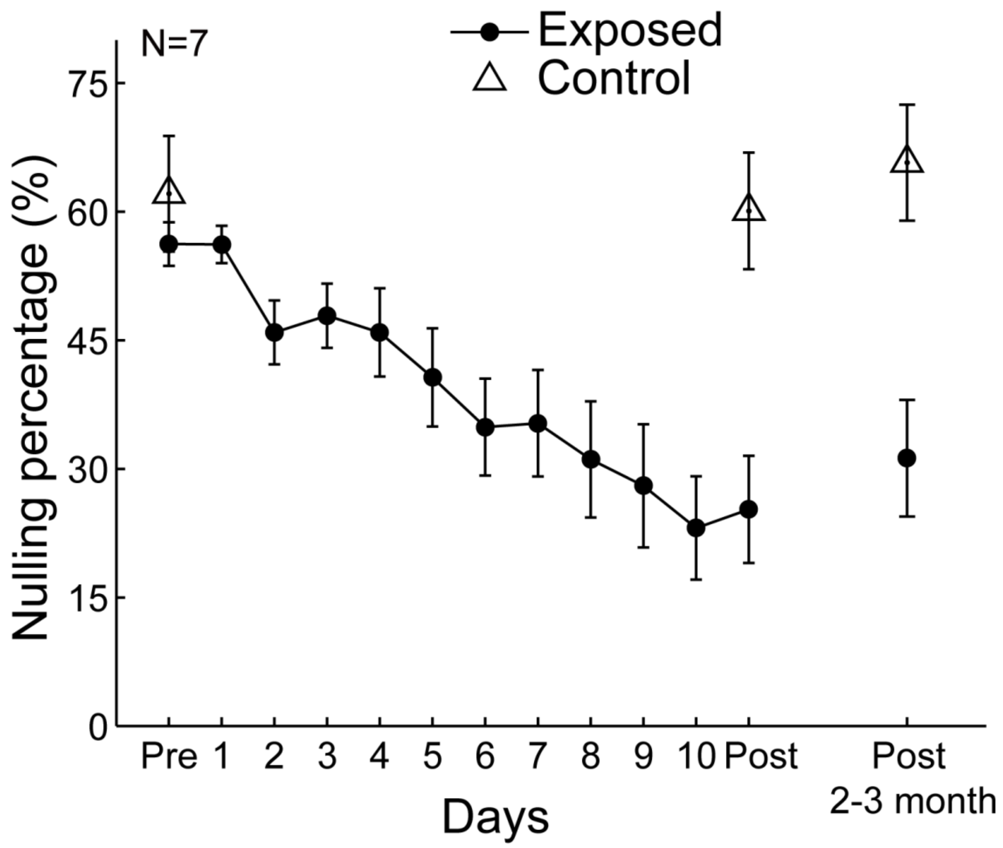

Figure S2. The training curve for the 7 subjects who participated in the follow-up test in Experiment 1.1. The training effects remained robust with only a small decay after 2-3 months. '•' denoted the exposed condition, while '△' for the control.

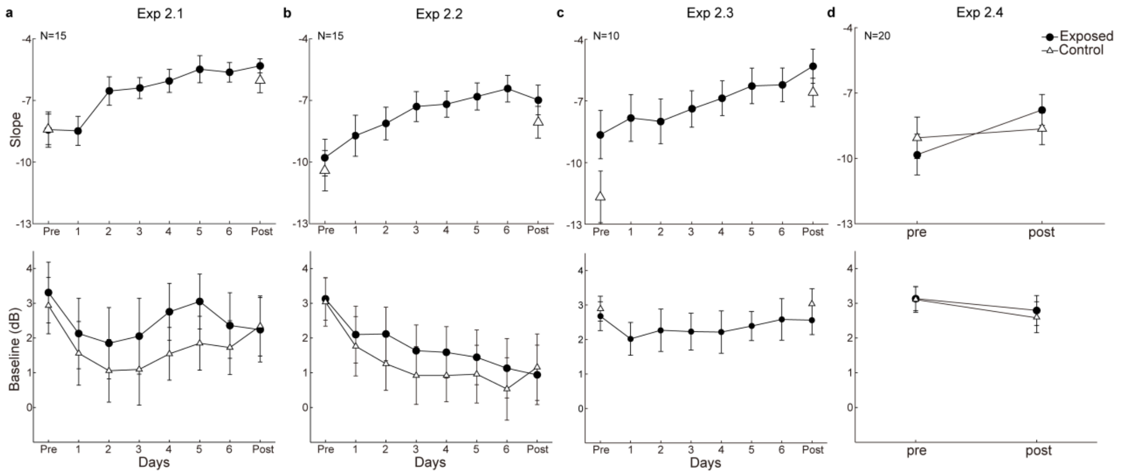

Figure S3. Slope of decay and baseline thresholds in the contrast adaptation experiments: The upper row showed the slope of decay in Experiments 2.1-2.4 where the ramp detection method was used. The lower row showed the changes of baseline thresholds for contrast detection over training (**a,b,c**) or before and after training (**d**). '•' denoted the exposed condition, while '△' for the control.

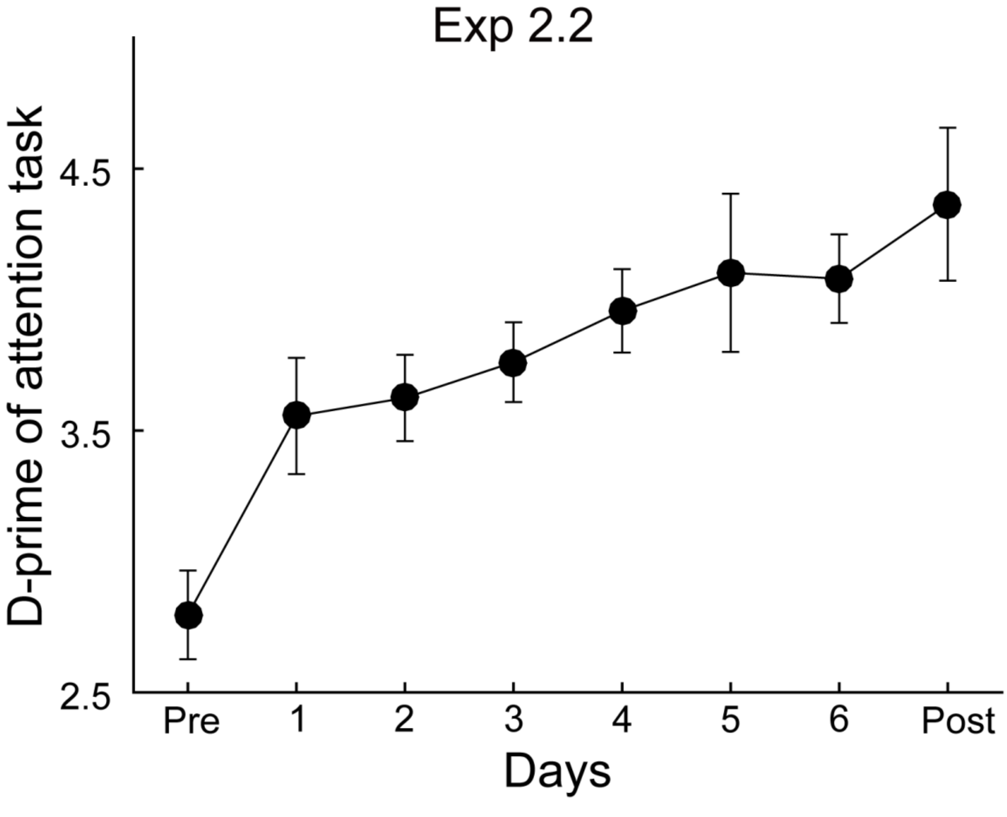

Figure S4. Training curve for the attention task in Experiment 2.2.

Supplemental References

1 Goldstone, R. L. Perceptual learning. *Annu Rev Psychol* **49**, 585-612 (1998).
